# *Drosophila muscleblind* Codes for Proteins with One and Two Tandem Zinc Finger Motifs

**DOI:** 10.1371/journal.pone.0034248

**Published:** 2012-03-29

**Authors:** Uwe Irion

**Affiliations:** Max Planck Institute for Developmental Biology, Tübingen, Germany; University of Florida, United States of America

## Abstract

Muscleblind-like proteins, Muscleblind (Mbl) in *Drosophila* and MBNL1-3 in vertebrates, are regulators of alternative splicing. Human MBNL1 is a key factor in the etiology of myotonic dystrophy (DM), a muscle wasting disease caused by the occurrence of toxic RNA molecules containing CUG/CCUG repeats. MBNL1 binds to these RNAs and is sequestered in nuclear foci preventing it from exerting its normal function, which ultimately leads to mis-spliced mRNAs, a major cause of the disease. Muscleblind-proteins bind to RNAs via N-terminal zinc fingers of the Cys_3_-His type. These zinc fingers are arranged in one (invertebrates) or two (vertebrates) tandem zinc finger (TZF) motifs with both fingers targeting GC steps in the RNA molecule. Here I show that *mbl* genes in *Drosophila* and in other insects also encode proteins with two TZF motifs, highly similar to vertebrate MBNL proteins. In *Drosophila* the different protein isoforms have overlapping but possibly divergent functions *in vivo*, evident by their unequal capacities to rescue the splicing defects observed in *mbl* mutant embryos. In addition, using whole transcriptome analysis, I identified several new splicing targets for Mbl in *Drosophila* embryos. Two of these novel targets, *kkv* (*krotzkopf-verkehrt*, coding for Chitin Synthase 1) and *cora* (*coracle*, coding for the *Drosophila* homolog of Protein 4.1), are not muscle-specific but expressed mainly in epidermal cells, indicating a function for *mbl* not only in muscles and the nervous system.

## Introduction

The *Drosophila* gene *muscleblind* (*mbl*) codes for a zinc finger protein that plays important roles during differentiation and development. The function of *mbl* is necessary for the differentiation of photoreceptors in the *Drosophila* eye and for development of larval and adult muscles [1], [2]. Mbl protein has been shown to bind to RNA and to regulate alternative splicing [3]. In *Drosophila* several muscle-specific transcripts, α*-actinin*, *ZASP52* and *troponinT*, are targets for regulation by *mbl* [4], [5].

In vertebrates there are three genes encoding orthologs of Mbl, *Muscleblind-like 1-3* (*MBNL1-3*) [6]. MBNL1 has been shown to play a crucial role in the pathology of myotonic dystrophy (Dystrophia myotonica, DM), an autosomal dominant disease, which is the most common form of muscular dystrophy affecting adults in humans. DM1, which accounts for the vast majority of the cases, is caused by an expansion of CTG repeats in the 3′-UTR of the DM protein kinase (DMPK) gene (reviewed in: [7]). Upon transcription the RNA containing these extended CUG repeats forms an unusual hairpin structure, to which MBNL1 protein binds [8], [9]. The binding of the protein to these toxic RNA molecules leads to the sequestration of MBNL1 in nuclear foci and ultimately to mis-regulated splicing of several transcripts, e.g. chloride ion channel (CLCN1), cardiac troponinT (cTNT) and insulin receptor (INSR) [10] [3] [11]. It is thought that the splicing defects are the main cause for the disease [12], [13].

In two mouse models for DM1, created either by a knockout of MBNL1 [14] or by over-expression of expanded CUG-repeats [15], similar splicing defects have been observed [12]. In addition it was found that over-expression of CUG-repeats leads to MBNL1 independent disruptions of extracellular matrix mRNA regulation, possibly regulated by MBNL2 [12].

MBNL2 was also shown to be involved in the localization of integrin α_3_ mRNA to focal adhesion sites in human cells [16].

MBNL proteins regulate alternative splicing by binding to intronic sequences in pre-mRNAs. Depending on the specific binding site, upstream or downstream of an exon, and most likely together with other factors, they promote the inclusion or exclusion of specific exons by antagonizing the activities of CELF proteins (CUG-BP and Etr-3-like factors) [17]. In cells and tissues from DM patients the observed splicing defects include retention of fetal exons, exon skipping, intron retention and the use of alternative splice sites [18].

The RNA binding motif for human MBNL1 is YGCY, where at least one pyrimidine base (Y) is U [19]. Intronic regions of genes regulated by MBNL1 are generally enriched for this motif. *In vitro* the *Drosophila* protein was found to preferentially bind to a five-nucleotide consensus sequence of AGUCU embedded in complex RNA secondary structures [20].

*Drosophila mbl* is a large gene, spanning more than 110 kb, which gives rise to several transcripts through alternative splicing [1], [2]. These transcripts encode proteins with a shared N-terminal region and different C-termini. Whereas vertebrate MBNLs contain two highly conserved tandem zinc finger (TZF) motifs in the N-terminal part of the protein, the fly proteins have so far mostly been described as containing only one TZF motif, as has the protein from the worm C. *elegans* [21]. Only recently a *Drosophila* transcript encoding a protein with two TZF motifs has been mentioned [18], [22], annotated in FlyBase [23] as transcript RF, but the conservation for the second TZF motif is considerably lower than for the first. Nevertheless it has been shown that *Drosophila* Mbl and human MBNL1 are functional homologs [24] and *Drosophila* has been used as a model organism to study the effects of RNA toxicity and the involvement of Muscleblind-like proteins in the pathology of DM1 [25], [26].

Here I show that the *Drosophila mbl* gene contains an additional, so far unknown, exon, which also codes for a second TZF motif. This exon is included in several splice-variants of the transcript. The additional, second pair of zinc fingers is of the characteristic Cys_3_-His type and highly similar to the second TZF motif in vertebrate MBNL proteins, making *Drosophila* Mbl more similar to its vertebrate orthologs than previously thought.

The different protein isoforms in *Drosophila* are likely to have different functions *in vivo*, as shown by their varying capacity to rescue the splicing defects observed in *mbl* mutant embryos.

In a global transcriptional analysis of *mbl* mutant embryos by RNAseq I find additional mis-spliced transcripts. The corresponding genes mainly code for cytoskeletal proteins with highest expression levels in muscles, but also for Chitin Synthase 1 (*kkv*) and for the septate junction protein Coracle (*cora*), which are expressed in epithelial cells.

These findings provide new insights into the *muscleblind* gene from *Drosophila*; and should ultimately also contribute to a better understanding of the regulation of alternative splicing in healthy and disease states in this important model organism.

## Results

### New splice variants from the *mbl* locus in *Drosophila*

**Figure 1:**
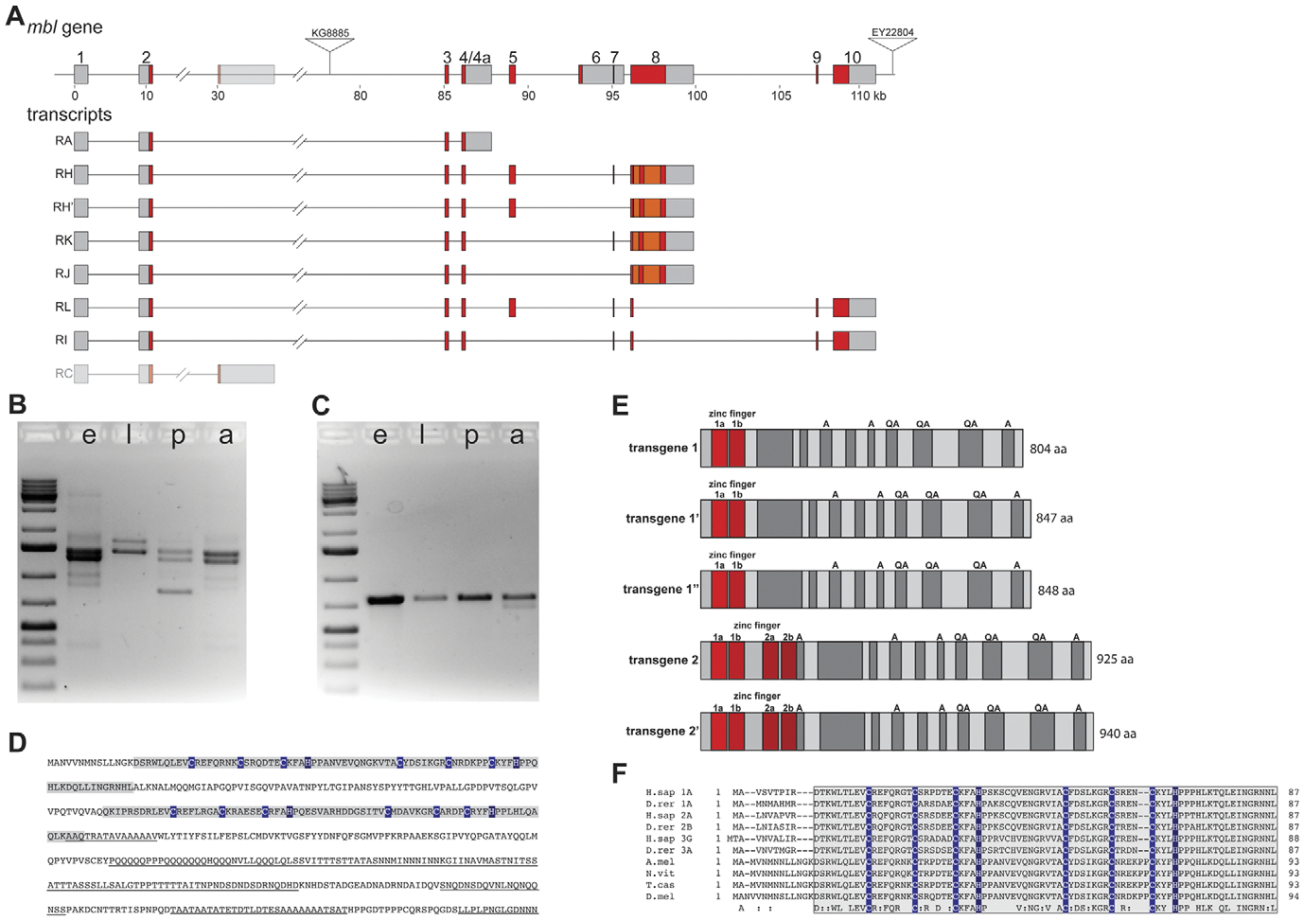
*Drosophila muscleblind* gene. **A** shows a schematic overview of the *mbl* locus in *Drosophila* and some of the annotated transcripts. Coding exons are in red and orange, non-coding exons in grey. The first TZF motif is encoded by exons 2 and 3, the second by the newly identified exon 5, which is included in some transcripts with downstream 3′ exons. Exon 8 often is only partially included in the final transcripts, the orange bits are sometimes spliced out. The insertion sites of the two P-Elements used to generate a small deletion are indicated (KG8885 and EY22804). **B** and **C** RT-PCR analysis of *mbl* expression during different developmental stages (e = embryos, l = larvae, p = pupae, a = adults). In **B** primers in exons 2 and 8 were used for the PCR amplification of cDNA fragments. Sequencing showed that the different bands correspond to differentially spliced transcripts with one TZF motif. In **C** primers in exons 2 and 5 were used to specifically amplify cDNA fragments with two TZF motifs; the identity of the 740 bp band was confirmed by sequence analysis. **D** amino acid sequence of the Mbl protein after translation of the longest identified ORF. The TZF motifs are delineated by grey boxes, the characteristic Cys and His residues are highlighted in blue. Stretches of low sequence complexity are underlined. **E** cartoons of the proteins encoded by the two rescue transgenes used in this study are shown. The zinc fingers are highlighted in red, stretches of low sequence complexity are shown in dark grey. Ala and Gln-Ala repeats are indicated. **F** shows the alignment of the amino-terminal parts of Mbl proteins from different species. The sequences show a high degree of similarity only in the zinc finger motifs (grey boxes). (H.sap = human, MBNL1A [GenBank: AAK94915.1], MBNL2A GenBank: [AAI04041.1], MBNL3G [GenBank: AAM09533.1]. D.rer = zebrafish, MBNL1A [GenBank: ABQ86048.2], MBNL2B [GenBank: ABR00800.1], MBNL3A [GenBank: ABX80004.1]. A.mel = honeybee, predicted from genomic sequence [GenBank: CM000054.5]. N.vit = jewel wasp, predicted from genomic sequence [GenBank: CM000916.1]. T.cas = red flour beetle, [NCBI Reference Sequence: XP_001812946.1]. D.mel = Drosophila melanogaster).

I identified an additional, previously not annotated, coding exon in the *muscleblind* gene of *Drosophila melanogaster* (exon 5 in Fig. 1A, genomic coordinates: 2R: 13241684..13241917, r5.36). The additional exon codes for a second TZF motif with very high similarity to the second TZFs from vertebrate Muscleblind-like (MBNL) proteins. In the latest version of FlyBase there are two transcripts containing this exon (RH and RL, see also Fig. 1). By RT-PCR a much larger number of different cDNAs containing this additional exon could be amplified. These PCR products roughly correspond to transcripts RH and RL, but I find a variety of differently spliced 3′-ends. For transcript RH in several cases varying parts of exon 8 are spliced out (Fig. 1A). Many of these transcripts are likely encode functional protein isoforms with different C-termini. In some cases, however, the observed splicing pattern leads to a frame shift followed by an early stop codon; the transcripts corresponding to these cDNAs might be artefacts. The shortest published isoform of *mbl* (RC, Fig. 1A [2]), which encodes a truncated protein of just 84 amino acids containing only part of the first TZF motif, could not be amplified by RT-PCR. Therefore, I suspect that this might not be a real splice variant of *mbl* but could have resulted from amplification of an intermediate, which is produced during the step-wise (recursive) splicing of the large (approx. 75 kb) second intron of the gene [27].

The cDNA with the longest ORF codes for a protein of 959 amino acids (Fig. 1D, [GenBank: JN812971]). This protein contains two TZF motifs in its amino terminal part but no other recognizable domains or motifs in the rest of the sequence, apart from several stretches of sequence with low complexity, some of them contain poly A (alanine), poly Q (glutamine) or poly QA repeats. Parts in the C-terminal region of the protein are predicted to be unstructured (disopred [28]).

By RT-PCR the expression of isoforms with one and with two TZF motifs could be detected during all stages of development (embryos, larvae, pupae and adults, see Fig. 1B and C). However, it was not possible to obtain expression patterns for the different isoforms by *in situ* hybridization; the differences between the isoforms with one or two TZF motifs are too small compared to the identical parts in the sequences.

BLAST searches revealed that other insect genomes also contain sequences with the potential to encode Mbl proteins with two TZF motifs. In general the genomic organization in other species is very similar to that of *Drosophila*. The intron positions are highly conserved from insects to vertebrates; the coding sequence for the first TZF motif is interrupted by a very large intron whereas the second TZF motif is encoded by one single exon. In the red flour beetle (*Tribolium castaneum*) the gene is located on linkage group 5, nt 8115648–8167288 [GenBank: CM000280.2]. In the yellow fever mosquito (*Aedes aegypti*) the sequences are found on supercont1.35 genomic scaffold, nt 2644576–3325965 [GenBank: CH477220.1]. In the jewel wasp (*Nasonia vitripennis*) the sequences are on chromosome 2, nt 4248516–4451760 [GenBank: CM000916.1]. In honey bees (*Apis mellifera*) the sequences are located on linkage group 1, nt 2648088–3394445 [GenBank: CM000054.5].

In the different insect species the size of the large intron between the first two coding exons ranges from approx. 40 kb in *Tribolium* to more than 180 kb in *Nasonia* and over 700 kb in *Apis*. The extraordinary large size of these introns in wasps and bees prompted further tests to verify the existence of the predicted transcripts. In both cases it was possible to amplify cDNA fragments of the expected size by RT-PCR on mRNA from pupae, using primers in the coding exons flanking the predicted large introns. Sequencing revealed that, indeed, the predicted cDNA fragments of 170 bp and 207 bp, had been amplified (data not shown).

The similarity between different vertebrate MBNL proteins and their homologs from insects is mostly restricted to the N-terminal zinc finger motifs (Fig. 1F). A comparison between *Drosophila* Mbl and human MBNL1A shows very high degree of conservation in the region of the zinc finger motifs, with 79% amino acid similarity for TZF motif 1 (67% amino acid identity) and 80% similarity for TZF motif 2 (61% identity). This is considerably higher than the similarity for the second TZF motif in MblF [18], the only other *Drosophila* Mbl protein published with two TZF motifs (32% identity, 49% similarity).

In the region between the TZF motifs there is only little conservation between human MBNL1 and *Drosophila* Mbl detectable. The similarities are mainly restricted to hydrophobic amino acid residues, and to Gln, Met and Pro residues. It has been suggested that this region is important for interactions with splicing co-regulators or spliceosomal components [29]. The only similarity in C-terminal parts of the proteins is the high occurrence of Ala and Gln residues.

### Regulation of alternative splicing by Mbl isoforms

*Drosophila* Mbl is known to affect alternative splicing of several muscle-specific transcripts, *troponin T* (encoded by the gene *upheld*, *up*) in pupae [5] and Z band associated transcripts (*Z band alternatively spliced PDZ-motif protein 52*, *ZASP52* and α-*actinin*, *Actn*) in embryos [4]. To analyze the functions of different Mbl isoforms in flies it would be advantageous to have a complete null-allele of *mbl*. The publicly available strong allele, *mbl^E27^*, is maybe not a complete loss-of-function allele, because some homozygous larvae still hatch from the eggs and die later [24]. Therefore I generated a new deletion-allele, *mbl^39/8^*, which removes almost the complete coding sequence of the *mbl* gene, leaving only exons 1 and 2 intact, coding for the first 64 amino acids of the protein. This was achieved by simultaneously mobilizing two P-elements, *P{SUPor-P}mbl^KG08885^* and *P{EPgy2}EY22804* (Fig. 1A, see Materials and Methods). The new allele leads to 100% embryonic lethality when homozygous or when over a deficiency for *mbl*, (*Df(2R)BSC154*). Both, hemizygous and homozygous embryos show a hyper-contracted abdomen, a phenotype characteristic for *mbl* mutants [24].

To test whether the different isoforms of the protein have different functions *in vivo*, I constructed several transgenes with upstream activating sequences (UAS) followed by coding sequences for different *mbl* isoforms, containing either one or two TZF motifs. This allows the expression of the different isoforms under the control of the GAL4 transcription factor. Altogether five different coding sequences were used for these transgenes. All of them were obtained by RT-PCR from embryonic mRNA, and they correspond approx. to transcripts RH, RH′, RJ and RK with differences in the splicing pattern of exon 8 (see Fig. 1A and E). Three of them have only one TZF motif, coding for proteins of 804 aa [GenBank: JN812966], 847 aa [GenBank: JN812967] and 848 aa [GenBank: JN812968]. Two of the transgenes encode longer proteins of 925 aa [GenBank: JN812970] and 940 aa [GenBank: JN12969] with two TZF motifs (Fig. 1E). The rescue ability of the different transgenes was assessed by expressing them in the null-mutant background, *mbl^39/8^* over *Df(2R)BSC154*, under the control of the ubiquitous driver *da::GAL4*. All five transgenes rescued the embryonic lethality caused by the mutation; but all of them failed to rescue completely, i.e. to adult viability. This is, however, most likely not an incomplete rescue of the *mbl* phenotype but rather a dominant phenotype due to ectopic (over-)expression of the *mbl* transcripts, because also the heterozygous mutant larvae expressing the transgenes did not survive.

**Figure 2:**
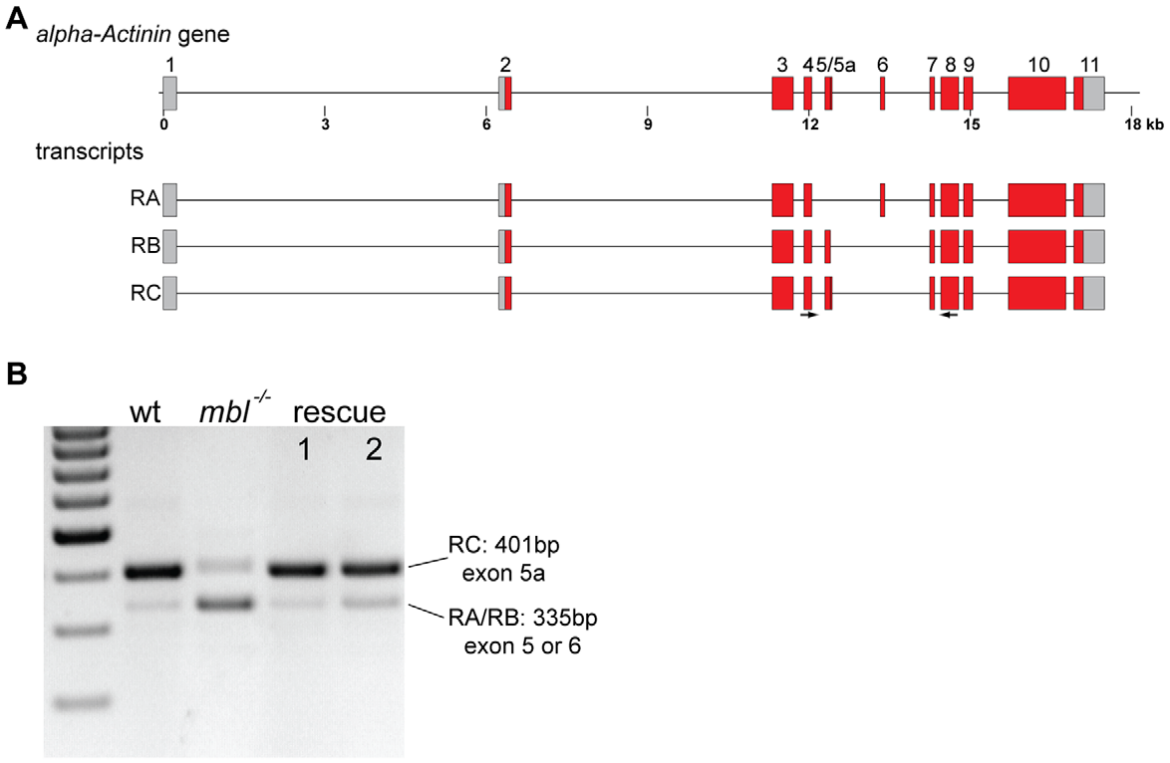
α*-actinin* alternative splicing and rescue. **A** schematic representation of the *Actn* gene in *Drosophila* and the three alternative transcripts, non-muscle isoform, RA, adult isoform, RB, and larval isoform, RC. **B** agarose gel showing the RT-PCR products generated using primers in exons 4 and 8 (arrows in a) from wild type embryos (wt), *mbl* mutant embryos (*mbl^−/−^*) and *mbl* mutant embryos expressing transgene 1 or 2 under the control of da::GAL4 (rescue 1 and 2). Two bands of 335 and 402 bp were generated, they correspond to transcripts RA or RB and RC. The marker is a 100 bp ladder.

I then tested the capacities of two transgenes, coding for isoforms with one (transgene 1, [GenBank: JN812966]) or two TZF motifs (transgene 2, [GenBank: JN812970]) (Fig. 1E), to rescue the splicing defects in *mbl* mutants. Mbl is known to be involved in the splicing of *Actn*, *ZASP52* and *up* transcripts in *Drosophila* [4], [5]. For *Actn* RT-PCR and sequencing showed that, in accordance with published data [4], in wild type embryos there are two splice isoforms present, they correspond to transcripts RA and RC (Fig. 2). RC, which is the predominant transcript, encodes an isoform specific for larval muscle, RA codes for the non-muscle isoform; RB, which encodes the adult muscle isoform, is not present. In *mbl* mutants there is a shift in the isoform ratio; whereas the abundance of the longer isoform, RC, is greatly reduced, the predominant form is now the shorter isoform (Fig. 2B); sequencing of the RT-PCR product revealed that both, RA and RB, are present in mutant embryos (data not shown). The *Actn* splicing defects in *mbl* mutant embryos are rescued by expression of either of the two transgenes, so that the longer isoform, RC, is again the most abundant splice variant in the embryos (Fig. 2B).

**Figure 3:**
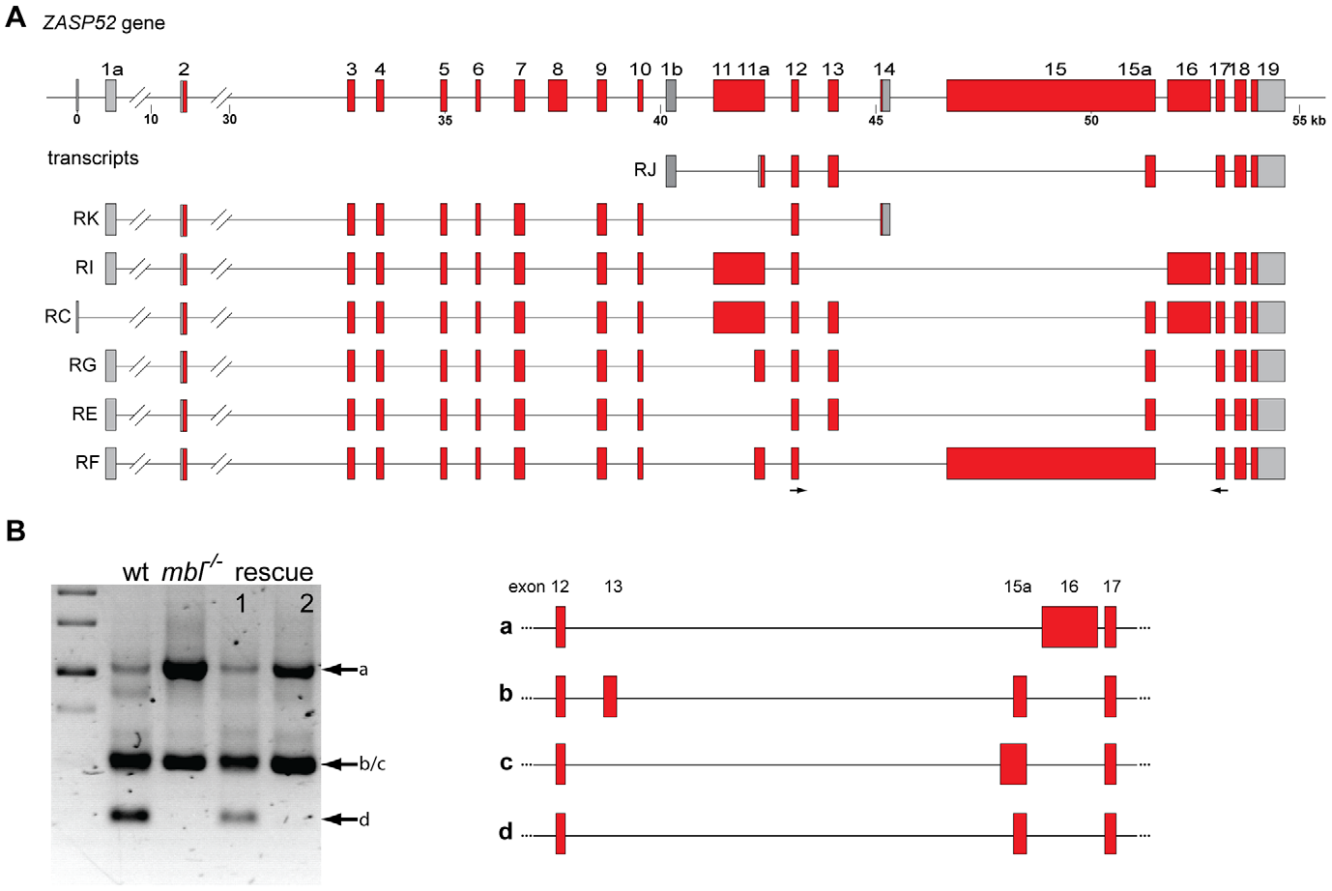
*ZASP52* alternative splicing and rescue. **A** schematic representation of the *ZASP52* gene in *Drosophila* and the alternative transcripts annotated in FlyBase. **B** agarose gel showing the RT-PCR products generated using primers in exons 12 and 17 (arrows in A) from wild type embryos (wt), *mbl* mutant embryos (*mbl^−/−^*) and *mbl* mutant embryos expressing transgene 1 or 2 under the control of da::GAL4 (rescue 1 and 2). Three bands of 304 and 520 bp and approx. 1 kb were generated, the corresponding splicing pattern is shown in the right panel, not all of them have annotated transcripts in FlyBase. The 520 bp fragment from the mutants is different from the fragment of the same size in wild type. The marker is a 1 kb ladder.

Alternative splicing leads to the production of several transcripts from the *ZASP52* gene. Similar to published data [4], RT-PCR from wild type embryos with primers in exon12 and exon17 produced two major bands of 304 and 520 bp and a minor band of approx. 1 kb. Sequencing of the these bands revealed the splicing pattern shown in figure 3 with exons 13, 15a and 16 being either excluded or included in the mature transcripts. In *mbl* mutants the longer form, where exon 16 is included, is more prominent (Fig. 3B), the shortest isoform is missing. There is also a form detectable that is not present in wild type, where a cryptic splice site within exon 15 is used (band c in Fig. 3B). In the case of ZASP52 only the expression of transgene 1 lead to a rescue of the defects and thereby to a splicing pattern very similar to wild type. Transgene 2, which codes for an isoform with two TZF motifs, did not alter the mutant splicing pattern significantly (Fig. 3B).

**Figure 4:**
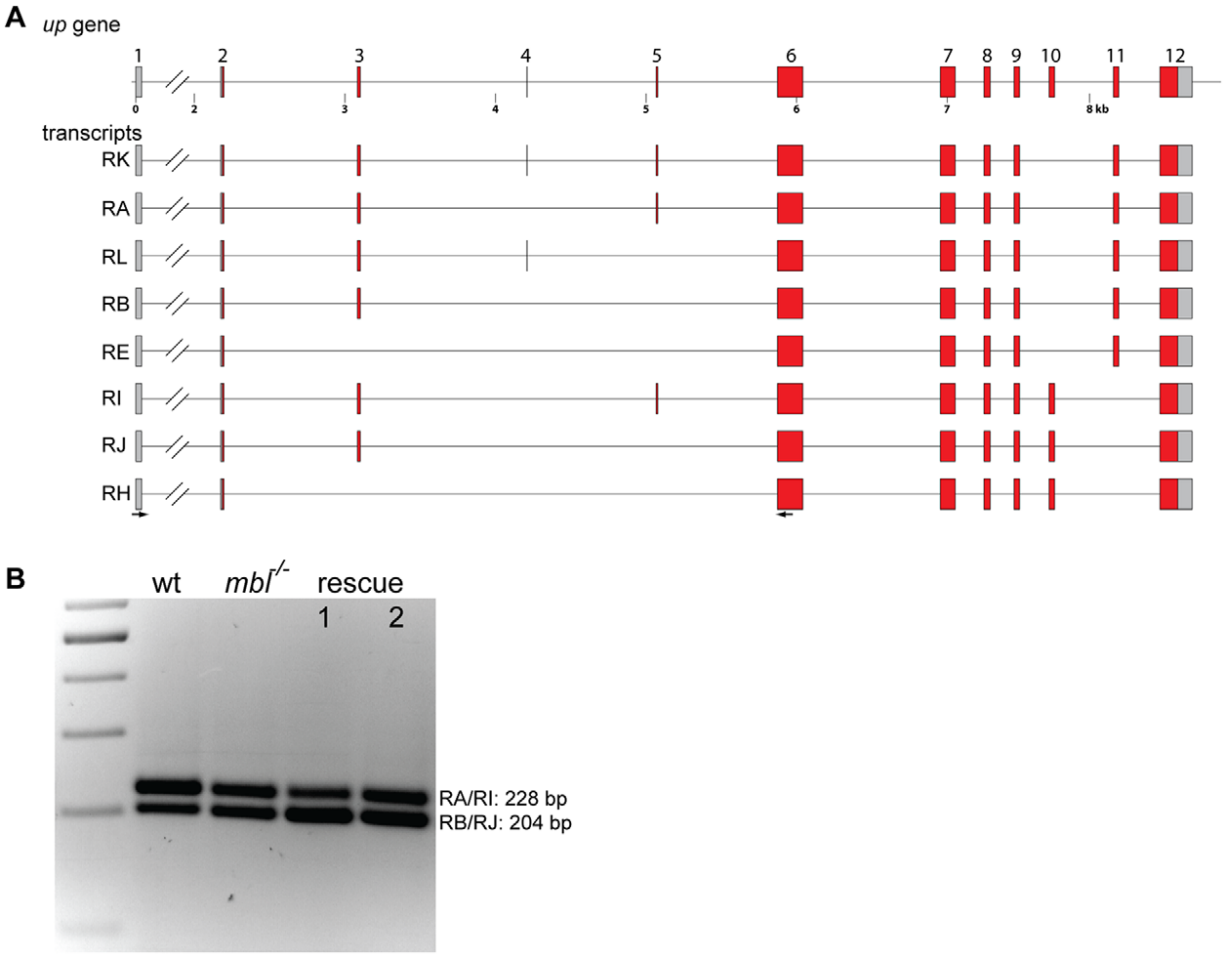
*upheld* (*up*, *troponinT*) alternative splicing and rescue. **A** schematic representation of the *up* gene in *Drosophila* and some alternative transcripts annotated in FlyBase. **B** agarose gel showing the RT-PCR products generated using primers in exons 1 and 6 (arrows in A) from wild type embryos (wt), *mbl* mutant embryos (*mbl^−/−^*) and *mbl* mutant embryos expressing transgene 1 or 2 under the control of da::GAL4 (rescue 1 and 2).Two bands of 203 and 227 bp were generated, they correspond to transcripts RB/RJ and RA/RI. The marker is a 100 bp ladder.

Splicing of transcripts from the gene *up*, which codes for the only TroponinT in the *Drosophila* genome, is affected in *mbl* mutant pupae [5]. In addition I found that also in mutant embryos the splicing of *up* is mis-regulated (Fig. 4). There is a shift in the ratio of the different isoforms. In the mutants there is a lower abundance of the longer isoform, with exon 5 included, as compared to the shorter isoform, where exon 5 is excluded (Fig. 4B). By sequencing the RT-PCR products I could not detect isoforms containing the very short exon 4 [30]. Neither of the two transgenes significantly rescued the splicing of *up* (Fig. 4B).

Sequencing of the RT-PCR products from *mbl* mutants frequently showed not only splicing defects by the inclusion or exclusion of specific exons but also defects in the splicing accuracy (data not shown).

### New targets for Mbl

To analyze the splicing defects in *mbl* mutant embryos on a genome wide level Illumina sequencing of the whole transcriptome of stage 16–17 embryos (RNA-seq) was performed. More than 33,000,000 short reads of 40 bases were obtained, which were aligned to the *Drosophila* genome using software from CLC Genomics Workbench. Analysis to find mis-spliced transcripts was carried out first by searching for transcripts with the highest number of exon-intron reads, and then by visually inspecting the top candidates on the list for potential splicing defects. 81 genes with more than 85 unique intron-exon reads could be identified (see Table S1). Amongst these genes are all the known targets of Mbl in *Drosophila*, *upheld* (*up*, *troponin T*), α*-actinin* (*Actn*) and *ZASP52*. The list is enriched for genes whose products are annotated with the GO terms for function: actin binding (11 genes), cytoskeletal protein binding (13 genes), protein binding (25 genes) and tropomyosin binding (3 genes). However, many genes on the list represent rather complex loci with multiple interspersed transcription units, or loci with (non-annotated) transposon insertions, which complicates the analysis, because in these cases the genome annotation might be incomplete and the assignment of intron-exon read could be wrong.

Ten transcripts were chosen for further, more detailed analysis by RT-PCR: *ZASP66*, *cora*, *wupA*, *Mf*, *CG31352*, *tmod*, *Mhc*, *CG33205*, *kkv* and *ssp4*. In five cases, *cora*, *wupA*, *Mf*, *CG33205* and *kkv*, differences in the splicing patterns between wild type and *mbl* mutant embryos could be confirmed.

**Figure 5:**
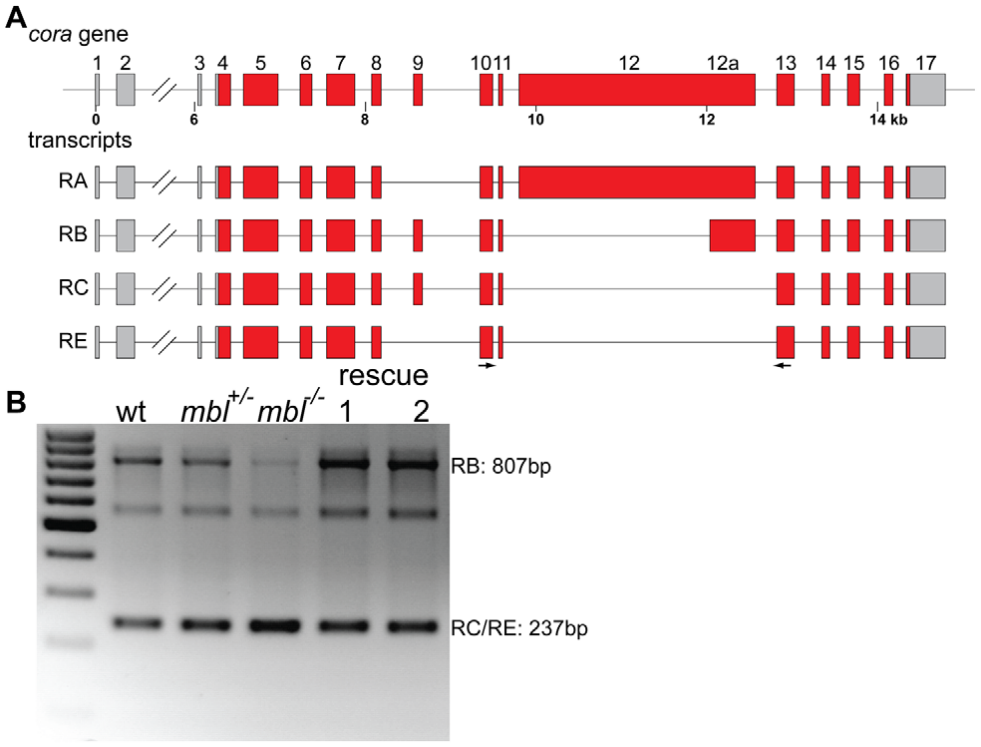
*coracle* alternative splicing and rescue. **A** schematic representation of the *cora* gene in *Drosophila* and the alternative transcripts annotated in FlyBase. **B** agarose gel showing the RT-PCR products generated using primers in exons 10 and 13 (arrows in A) from wild type embryos (wt), *mbl* mutant embryos (*mbl^−/−^*) and their siblings (*mbl^+/−^*) and from *mbl* mutant embryos expressing transgene 1 or 2 under the control of da::GAL4 (rescue 1 and 2). The two bands of 237 bp and 807 bp correspond to transcripts RC/RE and RB, respectively. The band of approx. 550 bp could be a different splice isoform or a background band from the PCR. The marker is a 100 bp ladder.

*coracle* (*cora*) codes for a FERM domain containing protein with homology to Band 4.1 from red blood cells [31]. In FlyBase there are four different transcripts annotated, these are produced by inclusion or exclusion of exon 9 in the mature transcript and by alternative splicing of exons12 and 12a (Fig. 5A). Using primers in exons 10 and 13, to test for the splicing of exon 12/12a, two major isoforms could be detected, one of them contains exon 12a, and corresponds to transcript RB, in the other one exon 12 is completely absent, which corresponds to transcripts RC and RE (Fig. 5B). In *mbl* mutants there is a clear shift in the isoform ratio with almost undetectable amounts of transcript RB. Expression of both rescue transgenes restored the wild type splicing pattern resulting again in a higher abundance of the longer isoform containing exon 12a (Fig. 5B). I also detected an additional band on the gel, which is not altered in the mutants. The attempt to sequence this band did not yield any results, it could be a PCR artefact.

**Figure 6:**
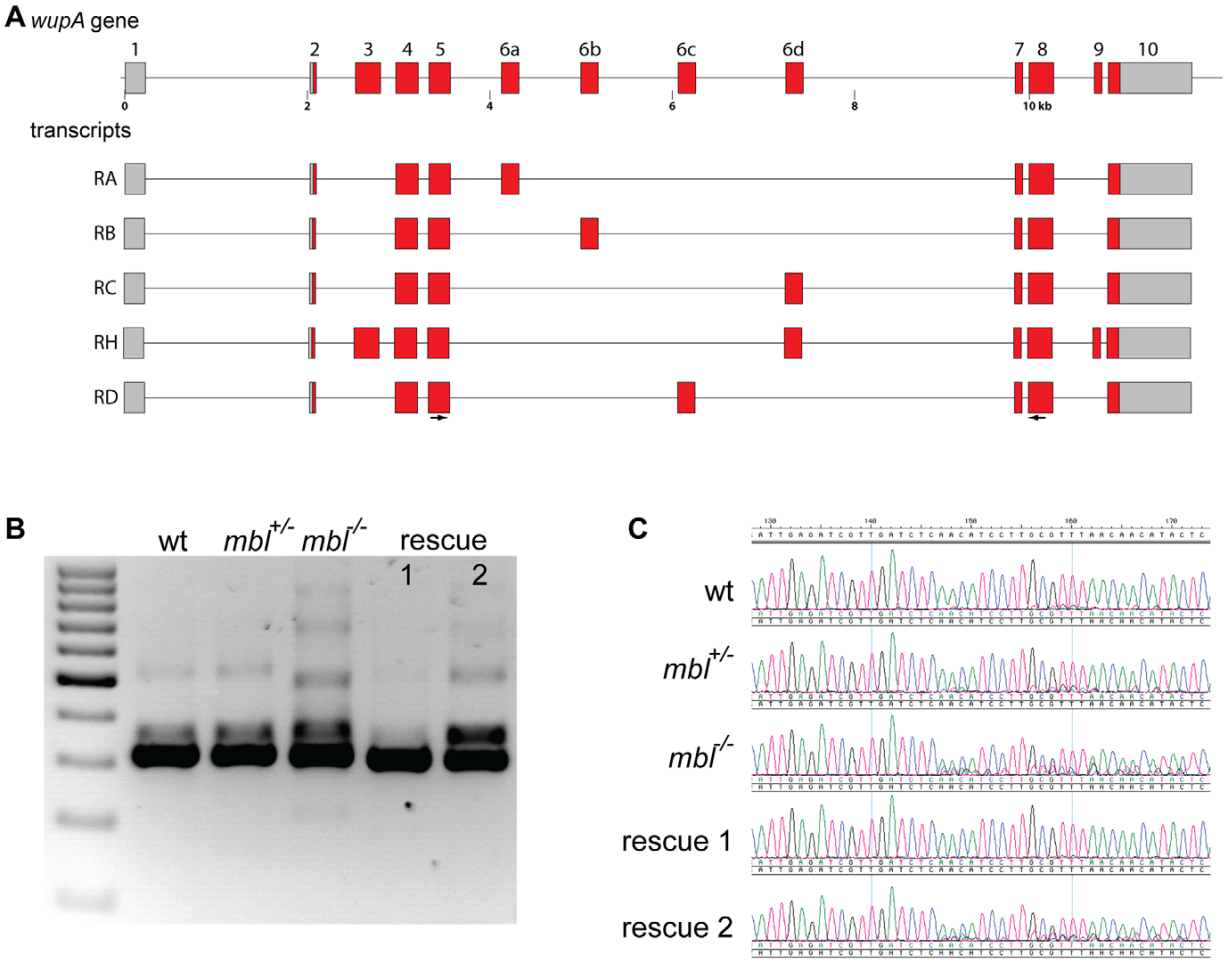
*wings up A* alternative splicing and rescue. **A** schematic representation of the *wupA* gene in *Drosophila* and some alternative transcripts annotated in FlyBase. **B** agarose gel showing the RT-PCR products generated using primers in exons 5 and 8 (arrows in A) from wild type embryos (wt), *mbl* mutant embryos (*mbl^−/−^*) and their siblings (*mbl^+/−^*) and from *mbl* mutant embryos expressing transgene 1 or 2 under the control of da::GAL4 (rescue 1 and 2). The marker is a 100 bp ladder. **C** sequence tracks from the RT-PCR bands showing a higher degree of heterogeneity in mutant embryos compared to wild type and rescue by transgene 1 but not by transgene 2.

In *Drosophila* the *gene wings up A* (*wupA*) encodes troponin I [32], which together with troponin C and troponin T forms the troponin complex that mediates thin filament response to calcium in striated muscles. There are ten different transcripts for *wupA* annotated in FlyBase, especially exon 6 is present in four mutually exclusive variants in the gene (exons 6a–d, Fig. 6A). I performed RT-PCR with primers in exon 4 and exon 8 (Fig. 6B) and then sequenced the resulting products. In wild type embryos the most abundant isoform corresponds to transcript RA, containing exon 6a, with a very low amount of transcript RB, where exon 6b is included, also being present (Fig. 6C). In homozygous *mbl* mutants a higher amount of sequences different from transcript RA is detectable (heterozygous peaks in Fig. 6C). Expression of rescue transgene 1 leads to a complete shift towards the inclusion of exon 6a whereas rescue transgene 2 did not affect the splicing significantly (Fig. 6C). Cloning of the RT-PCR products and sequence analysis of single clones showed, as expected, that transcript RA is the predominant form in wild type embryos (10 out of 12 clones). In homozygous *mbl* mutants only five out of 12 clones correspond to RA, the others correspond to RD or they contain both exons 6a and 6c. Transgene 1 rescues the splicing defects with RA being the dominant transcript again (11 out of 12 clones), whereas expression of transgene 2 leads to the lowest amount of RA (3 out of 12 clones).

**Figure 7:**
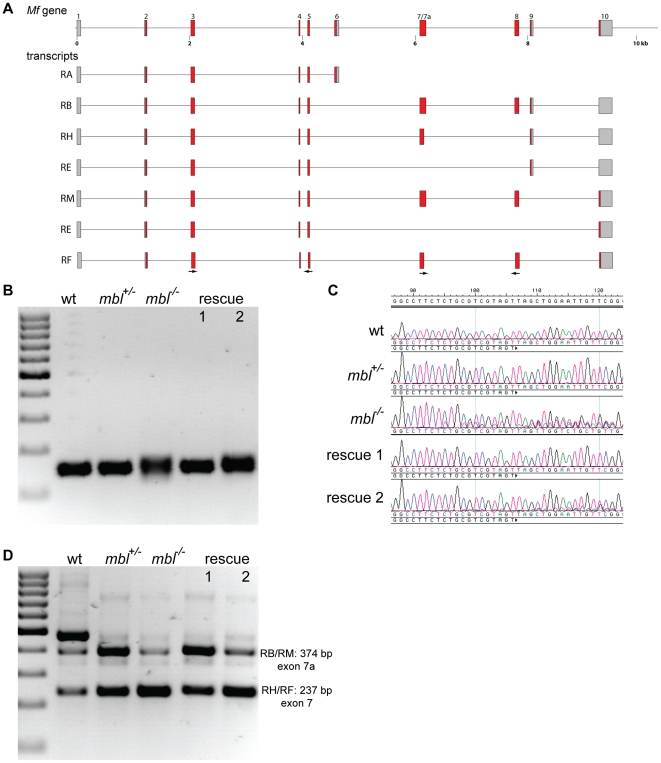
*Myofilin* alternative splicing and rescue. **A** schematic representation of the *Mf* gene in *Drosophila* and some alternative transcripts annotated in FlyBase. **B** agarose gel showing the RT-PCR products generated using primers in exons 3 and 5 (arrows in A) from wild type embryos (wt), *mbl* mutant embryos (*mbl^−/−^*) and their siblings (*mbl^+/−^*) and from *mbl* mutant embryos expressing transgene 1 or 2 under the control of da::GAL4 (rescue 1 and 2). The marker is a 100 bp ladder. **C** sequence tracks from the RT-PCR bands showing a higher degree of heterogeneity, due to lower splicing accuracy, in mutant embryos compared to wild type and rescue by transgene 1 and by transgene 2 to some extent. **D** agarose gel showing the RT-PCR products generated using primers in exons 7 and 8 (arrows in a) from wild type embryos (wt), *mbl* mutant embryos (*mbl^−/−^*) and their siblings (*mbl^+/−^*) and from *mbl* mutant embryos expressing transgene 1 or 2 under the control of da::GAL4 (rescue 1 and 2). The bands of 237 bp and 374 bp correspond to transcripts RF/RH and RB/RM. In wild type there is a strong band of 472 bp, which is due to the use of an alternative splice acceptor site for exon 8. The marker is a 100 bp ladder.

Myofilin (Mf) is a protein associated with the thick filaments in insect muscles [33]. According to FlyBase the *Mf* gene has 13 annotated transcripts; these are mainly produced by alternative 3′ exons and by the use of different splice donor sites in exon 7 (Fig. 7A). By RT-PCR several differences in the splicing pattern of *Mf* transcripts between wild type and *mbl* mutant embryos could be detected (Fig. 7B–D). In the mutants the accuracy of the splice acceptor site selection of exon 4 is lower, which leads to the production of two transcripts differing by 21 nucleotides. This effect is completely rescued by transgene 1 and partially rescued by transgene 2 (Fig. 7B,C). In addition, the relative ratio of transcripts with the short and long version of exon 7 is altered in the mutants (Fig. 7D). In wild type embryos both forms are easily detectable by RT-PCR; in the mutants, however, the longer form shows a much lower abundance. Transgene 1 rescues the defect and seems to push the ratio even more towards the longer form, whereas transgene 2 does not alter the mutant phenotype significantly. Surprisingly, only in wild type embryos an even longer isoform can be detected (Fig. 7D). Sequencing showed that this variant arises by the use of an alternative splice acceptor site of exon 8. However, this isoform could be non-functional or an artefact, because there is a stop codon soon after the new splice site; it was also not detected in heterozygous embryos, which develop like wild type, nor was it present in homozygous mutants or in embryos carrying the rescue transgenes.

**Figure 8:**
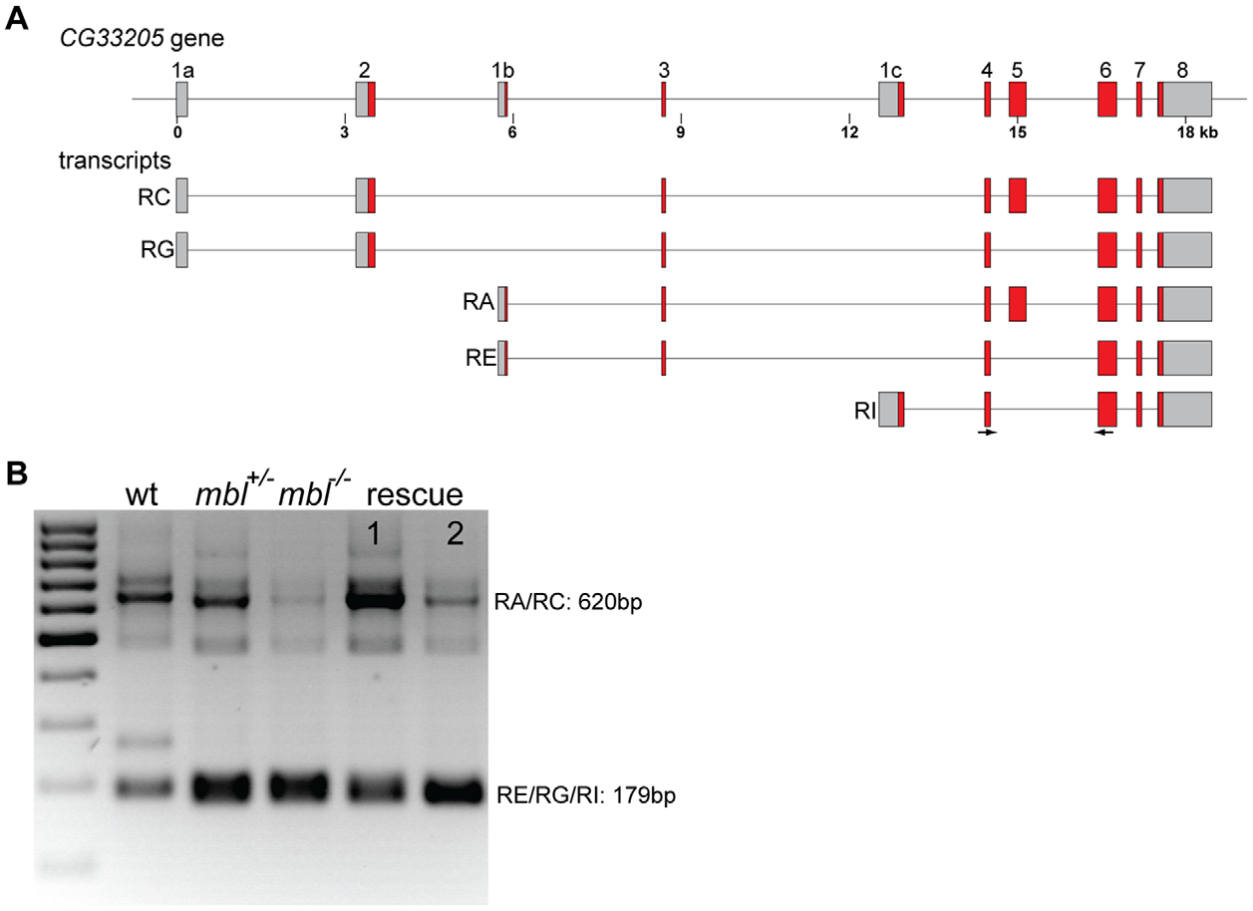
*CG33205* alternative splicing and rescue. **A** schematic representation of the *CG33205* gene in *Drosophila* and some alternative transcripts annotated in FlyBase. **B** agarose gel showing the RT-PCR products generated using primers in exons 4 and 6 (arrows in A) from wild type embryos (wt), *mbl* mutant embryos (*mbl^−/−^*) and their siblings (*mbl^+/−^*) and from *mbl* mutant embryos expressing transgene 1 or 2 under the control of da::GAL4 (rescue 1 and 2). The marker is a 100 bp ladder.

*CG33205* encodes a protein with a ZASP motif in its N-terminus. In FlyBase there are 8 transcripts annotated for this gene, they are produced by the use of alternative 5′ exons and by the inclusion or exclusion of exon 5 (Fig. 8A). I found that in *mbl* mutant embryos the longer isoform, corresponding to transcripts RA and RC, where exon 5 is included, is almost not detectable, whereas in wild type and heterozygous embryos there is a strong band of the expected size (Fig. 8B). Expression of rescue transgene 1 restored the wild type splicing pattern, maybe even lead to the production of more of the longer isoform, whereas rescue transgene 2 had almost no effect (Fig. 8B). Sequencing of the RT-PCR products revealed several length polymorphisms between heterozygous and homozygous *mbl* embryos (data not shown). However, these differences only affect the polyQ stretches in the C-terminus of the protein and are most likely not due to aberrant splicing, but rather to non-isogenic third chromosomes in the different embryos.

**Figure 9:**
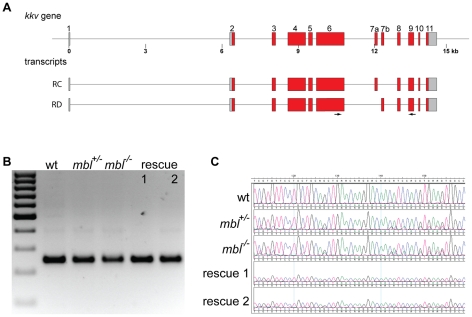
*krotzkopf verkehrt* (*Chitin synthase 1*) alternative splicing and rescue. **A** schematic representation of the *kkv* gene in *Drosophila* and two alternative transcripts annotated in FlyBase. **B** agarose gel showing the RT-PCR products generated using primers in exons 6 and 9 (arrows in A) from wild type embryos (wt), *mbl* mutant embryos (*mbl^−/−^*) and their siblings (*mbl^+/−^*) and from *mbl* mutant embryos expressing transgene 1 or 2 under the control of da::GAL4 (rescue 1 and 2). The marker is a 100 bp ladder. **C** sequence tracks from the RT-PCR bands showing a higher degree of heterogeneity in mutants and in heterozygous siblings compared to wild type embryos.

In *Drosophila* chitin synthase-1 is encoded by the gene *krotzkopf-verkehrt* (*kkv*) [34], which contains two mutually exclusive small exons of 176 bp (exon 7a and 7b, Fig. 9A). In wild type embryos the predominant form is the one where exon 7a is included, corresponding to transcript RC, with almost undetectable levels of the other form (Fig. 9B and C). In *mbl* mutants, and, surprisingly, also in heterozygous embryos, both isoforms are readily detectable in the sequences (Fig. 9 C). This splicing defect is rescued by transgene 1 and, to a lesser degree, by transgene 2 (Fig. 9C).

Taken together I found a number of transcripts with altered splicing patterns in *mbl* mutant embryos. These include the inappropriate use of alternative exons, shift in isoform ratios, and also more general defects in splice site selection. The two different transgenes I tested for their abilities to rescue the spicing defects in *mbl* mutant embryos show clear differences. Transgene 1, which codes for a protein of 803 amino acids with one TZF motif (Fig. 1C), rescues most defects, only the splicing of *up* is not rescued. Sometimes expression of this transgene seems to push the isoform ratio even more away from the mutant, e.g. for the *kkv* or *wupA* transcripts. Transgene 2, which differs from transgene 1 by an additional 121 amino acids encoding the second TZF motif, but has the same C-terminus, only rescues the splicing defects in *Actn*, in *cora* and, to some degree, in *kkv*.

## Discussion

### New isoform of Mbl in insects

*Drosophila mbl* is known to code for several proteins with one N-terminal tandem zinc finger motif; I found that the genes in *Drosophila* and in other insects (honey bee, wasp, mosquito) also encode protein isoforms with two TZF motifs, highly similar to the Mbl orthologs in vertebrates. The zinc fingers show a very high degree of conservation between insect Mbl and vertebrate MBNL proteins with almost 80% amino acid similarity. The only significant difference is the spacing between two Cys residues in the second zinc finger of the first TZF motif, with two additional amino acids in insect proteins.

Also, the genomic organization of the *muscleblind* genes is very similar, not only between insects, but also between insects and vertebrates. The intron positions are highly conserved and so is the large size of intron 2. This intron, which splits the coding sequence for the first TZF motif, spans 75 kb in *Drosophila* and, according to the latest genome assembly (Amel_4.5), more than 700 kb in honeybees. Nevertheless the spliced product could be easily detected in RNA from pupae.

In *Drosophila* transcripts for a large number of *mbl* isoforms coding for proteins with one or two TZF motifs are present during all stages of the life cycle. It proved to be difficult to estimate the relative abundance of the different types of transcripts. PCR primers designed to detect both types lead almost exclusively to the amplification of transcripts coding for only one TZF motif. This could be due to much higher abundance of these transcripts, however, it might also reflect a bias in the PCR amplification. Especially because primers designed to detect isoforms with two TZF motifs work very efficiently (see Fig. 1B and C).

There is no indication that vertebrates express MBNL proteins with only one TZF motif. However, for human MBNL1 and MBNL3 it has been shown that truncated versions of the proteins, lacking either one of the two TZF motifs, are still able to bind to RNA and to regulate splicing in a cell culture assay [29].

### Additional Mbl targets in *Drosophila*

To find additional targets for Mbl in *Drosophila* the complete transcriptome of stage 16–17 embryos hemizygous for a null-allele was analyzed by Illumina sequencing. Given the fact that I had detected a lower accuracy in splice site selection for the α*-actinin* and *ZASP52* transcripts in mutant embryos compared to wild type, I focused the analysis of the RNAseq data on the number of total and of unique intron-exon reads as a measure of splicing precision. The known Mbl targets in *Drosophila* are amongst the highest-ranking transcripts with more than 85 unique intron-exon reads. In the complete list of 81 candidates there is a clear enrichment for the GO annotation terms ‘actin binding’, ‘cytoskeletal protein binding’, ‘protein binding’ and ‘tropomyosin binding’ (Table S1), indicating the prominent function of *mbl* in muscle development and differentiation. However, many genes with a large number of intron-exon reads represent loci with multiple, often interspersed transcripts, which makes analysis very difficult, e.g. *Pde1c*, *Ect4* or *l(3)82Fd*. Some genes with many intron-exon reads also harbor transposons, which does account for some of the reads, e.g. *Cda5*.

Taking these limitations into consideration, the analysis revealed several new potential candidates as targets for Mbl in *Drosophila*. I tested ten of these candidates by RT-PCR and found splicing defects in five of them. The observed defects are either a shift in the ratio of different isoforms (*CG33205*, *cora*), the incorrect selection of mutually exclusive exons (*wupA*, *kkv*) or general defects in splicing accuracy (*Mf*).

### Possible functional differences between isoforms

An important question is whether the different proteins, with one or two TZF motifs and with different C-termini, have different functions *in vivo*. One possibility could be that all the different Mbl isoforms in *Drosophila*, which are generated from one gene by extensive alternative splicing, have similar and redundant functions regulating the alternative splicing of the same pre-mRNAs. Another possibility could be that the different proteins have different pre-mRNA targets. As a third alternative some isoforms could be involved in functions altogether different from the regulation of splicing, such as RNA localization. In vertebrates, MBNL proteins are encoded by three separate genes, and for MBNL2 a function in localizing the integrin α_3_ transcript has been demonstrated [16]. Whether any other Mbl protein besides MBNL2 is involved in RNA localization remains to be seen.

To address this question I tested the ability of Drosophila Mbl proteins with one or two TZF motifs to rescue the splicing defects occurring in homozygous mutant embryos. It has previously published that *Drosophila* Mbl regulates alternative splicing of two transcripts in embryos, α*-actinin* and *ZASP52* [4]. In homozygous mutant embryos the tissue specific and developmental timing dependent splicing of α*-actinin* is mis-regulated, e.g. with the premature occurrence of adult isoforms already during embryogenesis. Also in mutant embryos aberrant *ZASP52* transcripts can be detected, where a cryptic splice site in exon 15 is used. In both cases there was a clear rescue of the splicing defects by transgene 1, which encodes a protein with only one TZF motif. Transgene 2, which codes for a protein with both TZF motifs, only rescues the α*-actinin* splicing but not the splicing of *ZASP52*.

A third published target for Mbl in *Drosophila* is *troponinT* [5]. It has been shown that in mutant pupae alternative splicing of transcripts from this gene is mis-regulated. I find that also in embryos there is a shift in the ratio of two different isoforms of *troponinT* transcripts in *mbl* mutants. This defect is not rescued by either of the two transgenes tested.

Having identified additional transcripts mis-spliced in *mbl* mutant embryos, I tested again the two different transgenes for their ability to rescue these defects. There is generally good rescue with transgene 1 whereas transgene 2 only rescues the splicing of *cora* and *kkv* to some extent.

The observed differences between the two transgenes indicate that there might be functional differences between Mbl proteins with one and two TZF motifs. Both are clearly regulators of alternative splicing, as they efficiently rescue the splicing of α-Actinin, which also demonstrates that expression occurs from both transgenes and that the proteins function in the nucleus. The different rescue capacities for the other *mbl* targets could indicate different specificities of the protein isoforms, where the protein with two TZF motifs might have other, not yet identified, targets. An alternative explanation, which cannot be ruled out at this stage, is that the proteins are present in the cells in very different amounts or that they localize mostly to different sub-cellular compartments. Because both transgenes are inserted at the same genomic location on the X-chromosome and the same GAL4 driver-line was used it seems unlikely that significantly different RNA levels should be transcribed. Therefore one would have to assume that the translation efficiencies of the RNAs are different, or that the extra 121 amino acid residues affect the stability or localization of the protein encoded by transgene 2. This hypothesis will only be testable after generation of an antibody, which allows the detection of Mbl proteins at endogenous levels on western blots.

### Potential Mbl binding sites in intronic sequences

Apart from CUG repeats, the five nucleotide sequence, 5′-AGUCU-3′, has been identified as a consensus binding motif for *Drosophila* Mbl by *in vitro*-studies using a protein with the first TZF motif [20]. This sequence motif occurs, however, only rarely in intronic sequences flanking those exons that are mis-spliced in *mbl* mutants. It has also been suggested that Mbl recognizes complex RNA secondary structures, which would not be easy to predict.

On the other hand, it was found that expression of the human gene, MBNL1, can rescue embryonic lethality in *Drosophila*, suggesting that both proteins recognize the same target RNAs [24]. *In vitro* selection has lead to the definition of a binding motif for the human protein, which is a GC motif embedded in pyrimidines, 5′-YGCY-3′, with a preference for at least one pyrimidine-base (Y) being U [19]. In efficient high-affinity binding sites this motif often occurs in several copies in the RNA. In a simple model for the regulation of alternative splicing, exon exclusion is promoted when MBNL binds upstream of the exon, whereas binding downstream of the exon enhances its inclusion.

**Figure 10:**
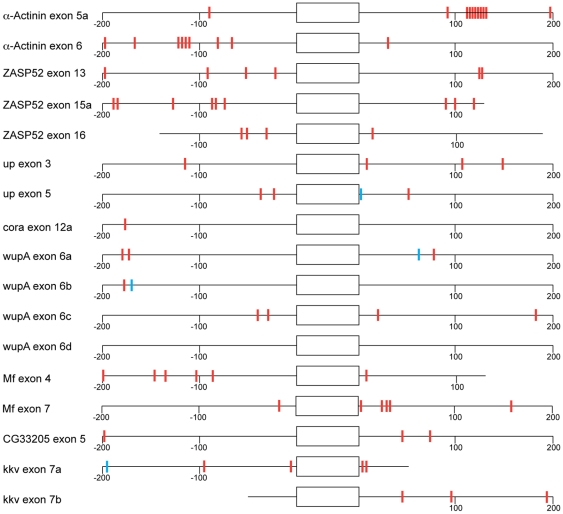
Distribution of YGCY motifs in intron sequences. Schematic representation of 200 bp intron sequences near exons that are mis-spliced in *mbl* mutants; for introns smaller than 400 bp only half of the intron sequence is depicted. YGCY motifs with at least one U are depicted as red boxes, the blue boxes represent AGUCU motifs, which have been described as binding sites for *Drosophila* Mbl. There is a clear accumulation of YGCY motifs in the neighborhood of alternatively spliced exons in α*-Actn*, *ZASP52*, *Mf* and *kkv*, for the other exons no enrichment above the overall background in the corresponding sequences from the complete *Drosophila* (1.72 YGCY motifs per 200 bp) genome is detectable. For simplicity the exons are not drawn to scale.

Given the high degree of similarity between the zinc finger motifs in *Drosophila* Mbl and human MBNL1 it seems likely that the *Drosophila* protein recognizes the same binding sites in pre-mRNAs. Analysis of the complete *Drosophila* genome showed that the binding motif occurs on average 1.72 times in intronic sequences close to exons (<200 bp) (Vipin T. Sreedharan and Gunnar Rätsch, personal communication). In the intronic sequences close to the exons 5 and 6 in *Actn* there are indeed clusters of YGCY motifs. In this case there is also good correlation with the model that Mbl binding downstream enhances inclusion (exon 5a) whereas binding upstream of the exon predominantly leads to exclusion (exon6). For the other exons where Mbl regulates splicing, mostly identified in this study, the correlation is less clear (Fig. 10). For example, in the case of *wupA* there is no clustering of YGCY motifs detectable, although the presence of Mbl protein clearly leads to the inclusion of exon 6a and the exclusion of exons 6b–6d. In several other cases it is not simple the inclusion or exclusion of an exon that is regulated by Mbl, but the selection between alternative splice sites for one exon.

*Drosophila* is an important model organism to study the function of Mbl proteins *in vivo* during development and their role in the pathogenesis of myotonic dystrophy. Here I show that the fly gene codes for proteins, which are more similar to the human proteins than previously recognized. The different protein isoforms seem to have distinct functions in the regulation of alternative splicing. Further analysis will be needed to understand the *in vivo* contributions of the different protein isoforms towards the regulation of alternative splicing and possibly in other processes.

## Materials and Methods

### *Drosophila* genetics

I used the following fly strains: *w^1118^* isogenized for the 2^nd^ and 3^rd^ chromosomes as wild type stock; the following flies were from the Bloomington *Drosophila* stock center: the stock carrying a GFP-marked 2^nd^ chromosome balancer: w*; *sna^Sco^/CyO*, *P{Dfd-GMR-nvYFP}2*; the strain carrying a chromosomal deletion for *mbl*: *w^1118^*; *Df(2R)Bsc154/CyO*; the two P-Element insertion strains: *y^1^; P{SUPor-P}mbl^KG08885^/CyO*; *ry^506^* and *y^1^ w^67c23^*; *P{EPgy2}EY22804*; the stock with the P-Element transposase source: *H{w^+mC^ = PDelta2-3}HoP8*, y^1^ w*; the stock for phiC31 integrase mediated transgene insertion on the 1^st^ chromosome: y^1^ M{3xP3-RFP.attP}ZH-2A w*; *M{vas-int.Dm}ZH-102D*; and the daughterless-GAL4 driver line: *w^1118^*; *P{w^+mW.hs^ = GAL4-da.G32}*.

To generate a small deletion, which removes most of the *mbl* coding sequence, *w^67c23^*; *P{EPgy2}EY22804* males were crossed to *H{w^+mC^ = PDelta2-3}HoP8*, y^1^ w*; *Pin/SM6a* virgin females and kept at 18°C to prevent premature mobilization of the P-Element as much as possible. In the next generation males of the genotype *H{w^+mC^ = PDelta2-3}HoP8*, y^1^ w*; *P{EPgy2}EY22804/SM6a* were crossed at 25°C to virgin females with the following genotype: *C(1)DX*, *y^1^w^1^f^1^*; *P{SUPor-P}mbl^KG08885^/SM1* to obtain males where both P-Elements will be mobilized simultaneously. These flies, with the genotype *H{w^+mC^ = PDelta2-3}HoP8*, y^1^ w*; *P{EPgy2}EY22804/P{SUPor-P}mbl^KG08885^*, were crossed to virgin balancer females of the genotype *w^1118^*; *In(2LR)*, *wg^Gla-1^ Bc^1^/SM1* and screened for white eyed males carrying the *SM1* chromosome in their offspring. These males were individually crossed to 3 to 4 balancer females. After they had produced offspring the male flies were tested by PCR for a deletion of the genomic sequence between the two P-Element insertion sites. The sequences of the primers were: Tue390 forward: CTTAAAACTGGTTACCCCCATTGC and Tue391 rev: CGTCAGCGACAAAGAGACGTAAAC. These primers are approx. 37 kb apart in the genome sequence of *Drosophila*, in the event of a precise deletion they produce a PCR product of 630 bp.

Transgenic flies were produced using the phiC31 system. The coding sequence for *mbl* was amplified by PCR, cloned into pCR2.1 and sequenced. Inserts coding for different isoforms were then transferred into pUAST-attB and the DNA was micro-injected into flies carrying a landing site on the 1^st^ chromosome and an integrase-source on the 4^th^, y^1^ M{3xP3-RFP.attP}ZH-2A w*; *M{vas-int.Dm}ZH-102D*. Transgenic offspring was identified in the F1 generation by the presence of the *white^+^* eye color marker.

### *mbl* RT-PCR

Trizol was used (Invitrogen) to extract total RNA from *Drosophila* embryos, L1 larvae, pupae and adults and from white pupae from honeybees (*Apis mellifera*) and from pupae of the parasitic wasp *Nasonia vitripennis*. After reverse transcription using oligo dT-primers and the Superscript II Kit (Invitrogen) the PCR was carried out with the following two primer pairs: *Drosophila mbl* for: GAATTCATGGCCAACGTTGTCAATATGAAC, *Drosophila mbl* exon8 rev: TGGCAGAGGTCGGTGCTCCGTAG, *Drosophila mbl* exon5 rev: CCGCTTTTAATTGTGCCTGTAGG; *A.mel* for.: AGGTTTGCAGAGAGTTCCAGCG, *A.mel* rev.: GCCCGTTTATCAAAAGCTGGTC; *N.vit* for.: ACCGGACACTGAGTGCAAGTTC, *N.vit* rev.: CGTTTATCAGGAGCTGGTCCTTC.

### RNA seq

To collect embryos hemizygous for the newly generated *mbl* null-allele (*mbl^39/8^*), flies of the following genotypes were crossed: w*; *Df(2R)BSC154/CyO*, *P{Dfd-GMR-nvYFP}2* and w*; *mbl^39/8^/CyO*, *P{Dfd-GMR-nvYFP}2* and from their offspring stage 16 to 17 embryos without YFP-signal were selected. From these embryos, *mbl^−/−^*, and from their siblings with YFP signal, for simplicity labeled *mbl^+/−^*, total RNA was extracted using Trizol (Invitrogen). mRNA was prepared from the total RNA with the Dynabeads mRNA DIRECT Kit (Invitrogen), following the manufacturer's instructions, including the optional re-extraction to eliminate rRNA contaminations. The library for the high-throughput sequencing was prepared using the mRNA-Seq. Sample Prep. Kit (Illumina) with a size selection for 250 bp, +/−50 bp, products. Sequencing was performed on an Illumina Genome Analyzer II instrument with 40 nt read length.

Analysis of the data was carried out using the CLC Genomics workbench software.

### RT-PCR analysis

Homozygous mutant embryos and their siblings were collected as described above. For the rescue experiment female flies with the genotypes: *M{w^+mC^ = mbl^rescue1^}ZH-2A*; *Df(2R)BSC154/CyO*, *P{Dfd-GMR-nvYFP}2* or *M{w^+mC^ = mbl^rescue2^}ZH-2A*; *Df(2R)BSC154/CyO*, *P{Dfd-GMR-nvYFP}2* were crossed to males with the genotype: w*; *mbl^39/8^/CyO*, *P{Dfd-GMR-nvYFP}2*; *P{w^+mW.hs^ = GAL4-da.G32}* and from their offspring embryos without CyO,YFP balancers were collected. In three independent experiments total RNA was extracted with Trizol and used for reverse transcription with oligo-dT primers and the Superscript II Kit (Invitrogen). The first strand reaction was diluted 1 to 10 and 1 µl was used as template in a 25 µl PCR reaction. The following primer pairs were used:

ACTN: forw.: CAAAGACAATCCTCTGGAGAATC, rev.: CACCTGCCAGCGAGTTGTCGGC. ZASP52: forw.: AAGGTGAACCAGGGCTATGCTC, rev.: CAATGCCGTGATAAAGGGTCC CG33205: forw.: GATCATCCGGGAGTTCACCC, rev.: GCTGTTGCTGTTGCCACTGTG. cora: forw.: TGCAGGAGCGGCTGTTAATG, rev.: TGCTGCTTGTTGGGATCGTG.

kkv: forw.: TCGACAATGATCCCGTGGAG, rev.: AACACCAATCCGATGGGCTC.

Mf(1): forw.: CCGTGCCATCTACGATCATCAC, rev.: TGAGTGGCTTGTCCCTCAAGTAG. Mf(2): forw.: CCGCTGGACGAACTCTTTGAAC, rev.: TGGGTGCTTGGACCACAAGTAG. wupA: forw.: GGCTGAACGTAGACGCATCATC, rev.: CTTCTTCACCACCTTGAGCTGG.

Touch down PCR was performed with the following cycling conditions 94°C for 2 min and then 10 cycles of 94°C for 30 sec, 60°C (−0.4°C/cycle) for 30 sec, 72°C for 1 min 20 sec, followed by an additional 25 cycles of 94°C for 30 sec, 56°C for 30 sec and 72°C for 1 min 20 sec, final extension 72°C for 3 min.

## Supporting Information

Table S1 **RNA-Seq analysis of** ***mbl*** **-mutant** ***Drosophila*** **embryos.** Output from CLC Genomics Workbench sorted for unique intron-exon reads (column L).
(XLSX)
